# Skin Regeneration, Repair, and Reconstruction

**DOI:** 10.1155/2015/892031

**Published:** 2015-07-21

**Authors:** Lars-Peter Kamolz, May Griffith, Celeste Finnerty, Cornelia Kasper

**Affiliations:** ^1^Medical University of Graz, 8036 Graz, Austria; ^2^Linköping University, 581 83 Linköping, Sweden; ^3^University of Texas Medical Branch, Galveston, TX 77555, USA; ^4^University of Natural Resources and Life Sciences, 1180 Vienna, Austria

Skin has important functions in several life-preserving processes such as hydration, protection against chemicals and pathogens, and heat regulation.

Severe damage to the skin may therefore be life-threatening. Skin regeneration and wound healing require an orchestrated integration of complex biological and molecular events, which include inflammation, proliferation, and remodeling. Despite the current use and availability of a wide array of wound dressings, ointments, and medical devices, wound healing still remains a clinical challenge, especially in the elderly, in diabetic patients, in heavy smokers, or in burned patients, because the time-consuming conservative wound management is mainly restricted to wound repair rather than restitution of the tissue integrity.

Therefore, there is a continued search towards more efficacious wound therapies to reduce health care burden and provide patients with long-term relief and ultimately scarless wound healing, because such wounds and defects if not treated effectively eventually end up in amputations or disfiguring scars termed as hypertrophic scars and keloids, because surgical procedures such as local or free flaps go along with limited donor site availability and require stable general health condition of the patients.

Therefore, there is a need of new strategies to promote wound healing and tissue repair. When talking of wound healing, a distinction is made between regeneration and repair. Regeneration is used to refer to the complete replacement of damaged tissue with new tissue not associated with scar tissue, while repair is used to refer to the reestablishment of tissue continuity. Regeneration can be attained by two means:

- restoration, defined as “putting together what is broken,”
- reconstruction, defined as “replacing and rebuilding what is torn down.”

To grant homeostasis, most tissues undergo continuous or cyclic processes of “degeneration” and regeneration. This special issue presents and compares different aspects of regeneration, repair, and reconstruction. By discussing the common traits and the specific features of regeneration, we propose general models of regeneration and highlight various strategies adopted to cope with damage and repair.

*Lars-Peter  Kamolz* *May  Griffith* *Celeste  Finnerty* *Cornelia  Kasper*

